# Biochemical and Botanical Aspects of *Allium sativum* L. Sowing

**DOI:** 10.3390/biotech11020016

**Published:** 2022-05-20

**Authors:** Ali Ammarellou, Ali Reza Yousefi, Moslem Heydari, Daniela Uberti, Andrea Mastinu

**Affiliations:** 1 Research Institute of Modern Biological Techniques, University of Zanjan, Zanjan 45371-38791, Iran; amarlou@znu.ac.ir; 2 Department of Plant Production and Genetics, University of Zanjan, Zanjan 45371-38791, Iran; m.heydari4066@znu.ac.ir; 3 Department of Molecular and Translational Medicine, University of Brescia, 25123 Brescia, Italy; daniela.uberti@unibs.it

**Keywords:** fresh weight, garlic, medicinal plant, pyruvic acid

## Abstract

The main aim of this study was to evaluate the yield and compliance of selected Iranian garlic (*Allium sativum* L.) cultivars, including Tuyserkan (TSN), Heydareh (HDH), Mouien (MUN), and Taroom (TRM), during two growing seasons. The TRM cultivar germination rate is higher than the other cultivars studied. The TRM cultivars have quite remarkable values for the dry weight, fresh weight, stem diameter, and the number of leaves present. The fresh weight and dry weight of the TRM cultivar for the second year are 33.8 t/ha and 16.7 t/ha, respectively. However, on average, the HDH cultivar is the tallest plant in the experiments. Average pyruvic acid content in fresh samples of the TRM and HDH cultivars is 78 µm/gfw and 69.3 µm/gfw, respectively. It is observed that there are remarkable differences in the level of pyruvic acid between the different cultivars. The growth, development, and yield of plants are highly dependent on their genetic characteristics; in this experiment, the TRM cultivar shows a good yield (16.7 t/ha), and the evaluated characteristics improve compared to the other cultivars studied, which could be due to the high compatibility of this cultivar to the environmental conditions of the study. The excellent performance on the yield of TRM makes this cultivar more appreciable on a commercial level.

## 1. Introduction

Garlic (*Allium sativum* L.) belongs to the *Alliaceae* family, and is a shallow-rooted vegetable crop [1,2]. *Allium* is an ancient crop that has its origins in central Asia and is widely distributed throughout the temperate, warm temperate, and boreal zones of the northern hemisphere [3,4]. A total of 7 out of the 750 known genus *Allium* species are cultivated in various areas of the world. Garlic belongs to the genus *Allium*, is one of the oldest cultivated vegetables, and the second most widely produced *Allium*, after onion (*Allium cepa* L.). Garlic has many uses, either as a raw vegetable (fresh leaves or dried cloves) for culinary purposes, or as a traditional and modern medicine ingredient [5,6]. Today, the use of medicinal plants is increasingly popular, and garlic cultivation increased around the world, due to its antioxidant properties [7,8,9]. The largest garlic producer in the world is China, which produces around 21 million tons of dry bulbs annually (over 81% of world production). The 25th most prolific garlic producer in the world is Iran, where 54.247 tons of dry bulbs are produced annually (FAO, 2013). Garlic has long had a special place in Iran’s traditional agricultural system because of culture, its medicinal properties, and adaptation to Iran’s climatic conditions.

Plants produce secondary metabolites used by humans for therapeutic, food, and aesthetic purposes [10,11,12,13,14,15,16,17,18,19]. Indeed, garlic’s main characteristic is the distinct flavor of its cloves, which is a result of complex biochemical reactions [20,21,22]. In particular, garlic flavor results after tissue disruption by the rapid catabolism of S-alk(en)yl-L-cysteine sulphoxide flavor precursors by the enzyme alliinase, which produces pyruvic, ammonia, and a range of both volatile and non-volatile sulfur compounds [23]. The main compounds in garlic are mostly sulfur-containing, non-volatile amino acids (thiosulfinates), among which alliin, or S-allyl-cysteine sulfoxide (ACSO), is the most predominant garlic flavor precursor [20]. Moreover, they may additionally increase the biosynthesis of glutathione, which shows important antioxidant functions [24]. Other significant volatile compounds with potent bioactive properties are ajoenes [25], as well as several sulfur-containing compounds other than alliin, such as allicin, 1,2-vinyldithiin, allicin, and S-allyl-cysteine [26,27]; and sulfides, such as diallyl-, methyl allyl-, and dipropyl mono-, di-, tri-, and tetra-sulfides, which are formed after the decomposition of thiosulfate [6]. 

Based on previous studies, it is determined that there is a positive correlation between flavoring compounds and pyruvic acid [28]. The enzymatic pyruvic acid represents the remaining capacity to produce flavor. The reduction in pyruvic acid content is attributed to the partial inactivation of alliinase, and the enzymatic and non-enzymatic destruction of flavor precursors [9,17]. Genetic diversity is an important component of biodiversity, and refers to inherited changes at the molecular level, and causes variation in DNA sequence, biochemical, and physiological properties [19,29]. Genetic diversity, referring to the diversity and variability between organisms, occurs at different levels among individuals in a population, species of the same genus, etc., and is a unique source in improving the genetic and breeding characteristics of plant products, as well as increasing the diversity of these products [30].

A study of Hirata et al. [31], on garlic cultivars worldwide, finds a variety of garlic phenotypes expressing a great diversity of traits, such as bulb weight, number of bulb per plant, bulb cover layers, leaf length, leaf diameter, number of leaves per plant, flowering ability, resistance to biotic stress, and resistance to abiotic stress [31]. The diversity in garlic cultivars provides an important basis for breeding and introducing new garlic varieties for effective use of genetic resources to improve breeding programs [31]. Garlic cultivars often have a specific physiological compatibility with specific agricultural and climatic conditions, resulting in an abundance of different cultivars [32]. Central Asia is the area with the highest number of different garlic germplasms and, consequentially, the area with the highest garlic diversity [33], but studies relating to garlic diversity were also conducted in different parts of the world, including the US, Latin America [34], Africa [35], and Europe [36]. Accordingly, it seems necessary to choose more compatible and high-yielding garlic cultivars for Iran’s climate conditions. Therefore, the main aim of this study was to evaluate the yield and compatibility of different Iranian garlic cultivars for the climatic conditions of Zanjan, Iran.

## 2. Materials and Methods

### 2.1. Site Description and Experimental Design 

The experiments were carried out in the Institute of New Biotechnologies’ (RINB) research farm, Zanjan University, Zanjan, Iran (35°25’ N, 47°1’ E), during the 2015/2016 and the 2016/2017 growing season. The region is characterized by a Mediterranean climate, with cold winter. The 30years annual mean temperature and precipitation were 11.5 °C and 419 mm, respectively. The soil texture was loam, with a pH of 7–7.3, to a depth of 0.5 m (Table 1). The experiments were arranged in a complete block design with 3 replications per treatment.

### 2.2. Experiment Procedure

Four certified garlic cultivars were ordered from Hamedan Garlic Research Center (HGRC) Hamedan, Iran. The cultivars ordered were Tuyserkan (TSN), Heydareh (HDH), Mouien (MUN), and Taroom (TRM). Twenty healthy, marketable, and same-sized garlic bulbs (each bulb containing 6–8 cloves) were randomly selected and their cloves separated, thenabout 80 healthy same-sized cloves were selected from the 100–150 cloves obtained from the bulbs. These cloves were randomly planted in rows (each block consists of four rows). The rows were 35 cm apart, and the plants in each row were 15 cm apart. The cloves were planted at a depth of 5 cm. The plants were drip irrigated twice, with an interval of ten days, and other field operations were performed as needed during the growing season. From April to harvest time (July), drip irrigation was performed every week, and weeding was done manually. No chemical fertilizers or pesticides were used.

### 2.3. Measurement of Traits

The morphological and yield characteristics were measured at the different growth stages using the standard descriptors for garlic development [37], including the days to germination, stem diameter, plant height, number of green leaves, fresh weight, and dry bulb yield after harvest. An equal number of garlic bulbs for each cultivar were selected and stored at room temperature and under shade. Germination was recorded for all cultivars over a 70day period (after which germination had occurred in all the experimental units). The physical and biological characteristics of the cultivars were evaluated six months after harvest and storage. The terminal meristem length and the number of buds were measured for each bulb cultivar. Pyruvic acid (PA) was also measured, using the method of Ketter and Randle [38].

### 2.4. Statistical Analysis

Data were subjected to variance analysis (ANOVA) using SAS software (version 9.1) and Microsoft Excel 2013. If the analysis of variance indicated statistically significant differences, the means were compared using the Duncan’s multiple range test (*p* < 0.05).

## 3. Results and Discussion

In order to evaluate the organoleptic parameters of the *Allium* cultivars, the nutritional conditions of the soil in the two growing seasons are evaluated (Table 1). The soil potassium content drops dramatically from 302 mg/kg in 2015/16, to 120 mg/kg in 2016/17. Likewise, the nitrogen content is low in both seasons (less than 1%). On the contrary, the phosphorus content in both years is optimal, around 20 mg/kg in 2015/16, and 17 mg/kg in 2016/17. This nitrogen deficiency in the two years may negatively affect the yield of *Allium* cultivars, despite the availability of other nutrients in the soil as already reported by other authors [14,39,40,41,42,43,44,45,46,47,48,49,50,51,52,53]. Subsequently, the processes of germination, development, and yield of the *Allium* cultivars are evaluated.

Complete germination time is significantly dependent on the type of cultivar, which, in the case of the TRM cultivar, is 40–45 days after sowing(DAC), while the TSN cultivar needs 63–65 days to complete germination (Table 2). All bulbous vegetables have a slow growth stage after germination [3]. The success of plant colonization and establishment is critically related to germination [54], and is expected to be variable according to genetic and geographical conditions [55]. The ability of *A. sativum* to germinate in this study (Table 2) may be due to the genetic makeup and climate adaptability [56] of the TRM cultivar. Major differences in germination occur in 2015–16 [57,58,59]. However, in this study, it is found that differences in germination are also significant in 2016–17 (Table 2).

The results illustrate that there is a high variation in garlic plant height over cultivars in both years (Table 2). The highest heights (63.3 cm and 65 cm) are observed for the HDH cultivar and the lowest (50.3 cm and 49 cm) for the TRM cultivar in 2015–16 and 2016–17, respectively (Table 2). Stem diameter decreases with increasing plant height. The TRM cultivar has the thickest stem (1.6–1.7 mm) for both years (Table 2).

One of the most important characteristics of bulbous plants is a strong and thick stem, able to transfer the photosynthetic compounds produced in the shoot and the photosynthetic organs quickly and easily to the roots. The high variation in the stem diameter and the number of leaves indicates the potential to develop leafy garlic variations for fresh consumption, since fresh garlic leaves are in great demand in some countries [6]. Our data show thatthe cultivars selected to produce more leaves are MUN and, especially, TRM. Indeed, the final number of leaves on a plants is a complex variable, and dependent on plant genetic makeup that codifies for different elementary processes, such as cell production, cell expansion, duration of leaf expansion, rate of leaf expansion, and leaf production rate [60]. The TRM cultivar produces the highest number of leaves (6.3 N°/plant) for both years (Table 2). The TRM cultivar produces, on average, 5.6 and 6.3 leaves per plant for the first and second year, respectively. This might be attributed to the TRM cultivar having a higher genetic potential in leaf production than other cultivars.

**Figure 1:**
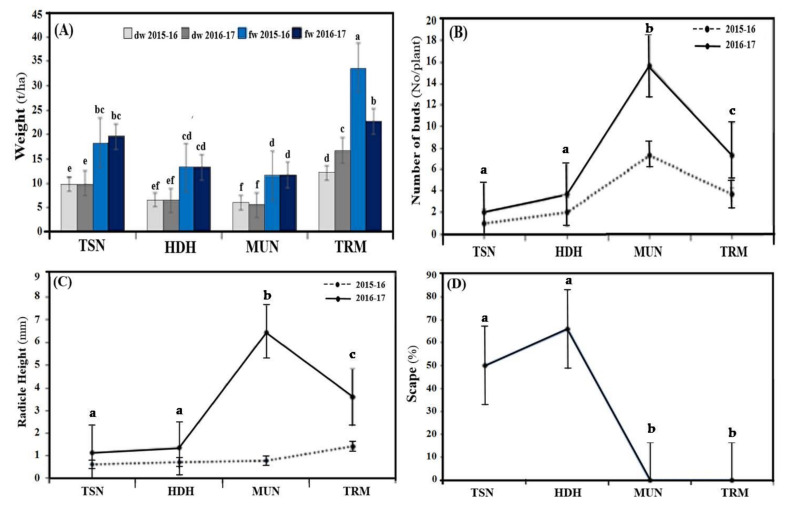
(**A**) Dry weight (dw) and fresh weight (fw), (**B**) number of buds, (**C**) radicle height, and (**D**) scape of *A. sativum* in different cultivars. Data are shown as mean ± standard deviation, and different letters denote statistically significant differences between experimental groups at the *p* < 0.05 level, according to Duncan’s multiple range test.

Regarding bulb yield (in terms of weight, Figure 1A), in all experimental groups, the 2016/2017 growing season produces larger bulbs than the 2015/2016 growing season. Moreover, the TRM cultivar has the highest bulb yield for both years (Figure 1A). In particular, TRM reaches about 35 t/ha in 2015/2016, and about 25 t/ha in 2016/2017. The increase in the TRM cultivar bulb yield might not only be ascribed to its genetic potential, but also to the higher vegetative indices, such as the number of leaves and stem diameter (1.6 and 1.7 in the two growing seasons, Table 2). Indeed, optimal development of leaves and stemleads to higher levels of photosynthesis and, consequently, the transfer of higher quantities of nutrients from the leaves to the bulb. The higher yield of garlic due to irrigation conditions may be caused by several factors, such as nutrition [61]. Moisture increases the RWC, stomatal conductance, carbon assimilation, stem volume, and, consequently, leaf volume, which absorb more radiation and increase the activity of photosynthetic enzymes, resulting in an increase in photosynthesis [62], and an increased yield [62,63,64] of garlic.

The quality of garlic products is usually assessed on the basis of their sensory attributes, mainly color, pungency, or flavor strength. The characteristic flavor of onions and garlic is attributed to the sulfur-containing volatiles. The formation of these volatiles is due to enzymatic reactions catalyzed by *alliinase,* and is accompanied by pyruvic acid production [65,66,67,68]. Variations in the enzymatic pyruvic acid (PA) content of garlic are illustrated in Table 2. It is observed that there are remarkable differences in the level of pyruvic acid between the different cultivars. It must be noted that the enzymatic pyruvic acid has the function of representing the remaining capacity to produce flavor. The TRM cultivar shows the highest levels of PA in the two seasons (79.3 µm/gfw in 2015/16, and 78 µm/gfw in 2016/17). The lowest levels of PA are observed in HDH in the two growing seasons, 71 µm/gfw and 69 µm/gfw, respectively. The reduction in PA content can be attributed to the partial inactivation of *alliinase* in some *Allium* cultivars, and to the enzymatic and non-enzymatic destruction of flavor precursors [22,66].

The genotypes are divided according to the different plant characteristics. All the cultivars have a medium number of bulb buds. However, the greatest number of buds are observed in the MUN cultivar (Figure 1B). In particular, MUN reaches around 6 buds per plant in the 2015/16 season, and around 16 in the 2016/17 season.

Regarding the development of the radical (Figure 1C), it is found that during the 2015/16 growing season, no significant differences are observed between the cultivars studied. In particular, the radicle does not have an extension greater than one millimeter. On the contrary, in the 2016/17 season, the TRM and MUN cultivars develop a longer radicle, about 6 mm and 4 mm, respectively.

**Figure 2:**
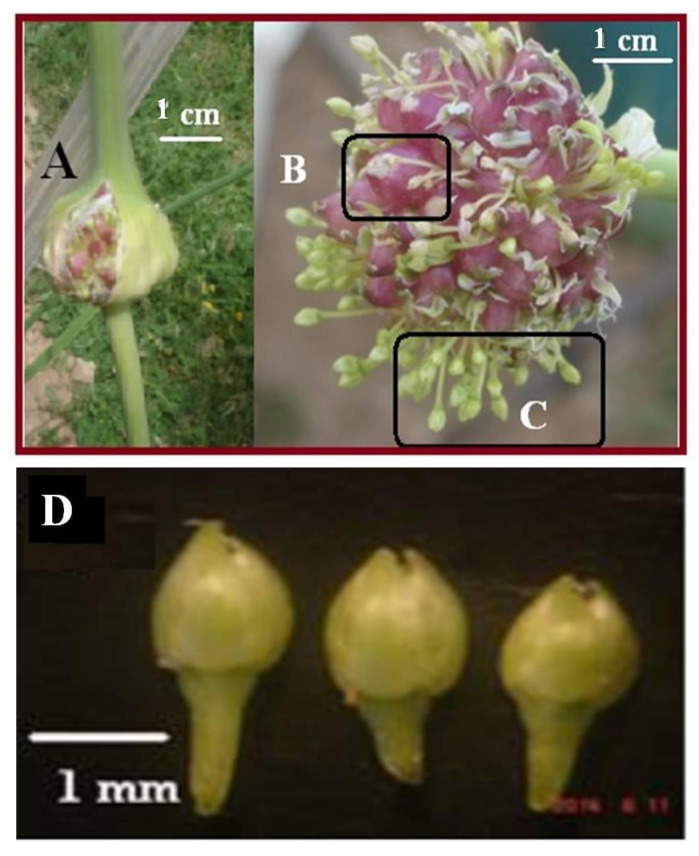
(**A**) Composite flowers of garlic, (**B**) aerial bulb, (**C**) barren florets of garlic, and (**D**) garlic flower structure.

The four garlic cultivars studied are divided into two main morphological groups, according to the ability to produce scape: the first group presents two cultivars that produce scape (bolting of garlic), and include the TSN and HDH cultivars; the second group has two cultivars that do not produce scape (non-bolting garlic), and is composed of the MUN and TRM cultivars (Figure 1D). The scape is produced only in the 2016/17 season, reaches 50% in TSN, and exceeds 60% in HDH. Based on the results, the storage life of the TSN and HDH cultivars is longer than the TRM and MUN cultivars (Figure 2).

## 4. Conclusions 

In this study, the germination rate of the TRM cultivar is higher than that of the other cultivars. The stem diameter and the number of leaves of the TRM cultivar are superior to those of the other cultivars for both years studied. However, on average, HDH cultivars produce the tallest plants during the experiment. The TRM cultivar shows significant values in terms of dry weight, fresh weight, and pyruvic acid content. Pyruvic acid contributes to the pungent aroma of *Allium sativum,* and its variations are associated with the partial inactivation of alliinase. Since dry weight, fresh weight, and stem diameter are very important indices for the evaluation of garlic yield, it is concluded that the TRM cultivar has a significant advantage in its diffusion, over other cultivars.

In conclusion, the phenotypic and genotypic diversity of garlic cultivars leads to large variations in traits such as bulb weight, number of bulbs per plant, bulb cover layers, leaf length, leaf diameter, and number of leaves. This diversity in garlic cultivars provides an important basis for the breeding and introduction of new garlic varieties. Furthermore, the assessment of garlic germplasms leads to the effective use of genetic resources to improve breeding programs.

## Figures and Tables

**Table 1:** The soil characteristics of experiment site.

Specifications	Depth (cm)	pH	EC * (dS/m)	Texture	N (%)	K (mg/kg)	P (mg/kg)
2015–16	10–50	7	0.9	loam	0.5	301.8	20.33
2016–17	10–50	7.3	1.3	loam	0.81	119.7	16.8

* EC = electrical conductivity.

**Table 2:** Germination rate, plant height, stem diameter, number of leaves, and enzymatic pyruvic acid of *Allium sativum* in different cultivars.

	Germination Rate		Plant Height		Stem Diameter		N° of Fresh Leaves		Pyruvic Acid	
	(DAP *)		(cm)		(mm)		(n/plant)		(µm/gfw)	
	2016	2017	2016	2017	2016	2017	2016	2017	2016	2017
TSN	65.6 ^a^	63 ^a^	55 ^b^	56 ^b^	1.4 ^a^	1.4 ^b^	4.3 ^b^	5 ^b,c^	74 ^b^	70 ^c^
HDH	62.3 ^a.b^	61 ^a^	63.3 ^a^	65 ^a^	1.06 ^b^	1.2^c^	4.3 ^b^	4.6 ^c^	71 ^c^	69 ^c^
MUN	60 ^b^	62 ^a^	53.3 ^b^	54 ^b^	1.5 ^a^	1.5 ^a.b^	5.6 ^a^	6 ^a,b^	73.6 ^b^	73 ^b^
TRM	43.3 ^c^	40 ^b^	50.6 ^b^	49 ^b^	1.6 ^a^	1.7 ^a^	5.6 ^a^	6.3 ^a^	79.3 ^a^	78 ^a^

* DAP = day after planting, fw = fresh weight. The superscript letters indicate the differences between the different treatments (*p* < 0.05) using the Duncan’s test.

## Data Availability

The data presented in this study are available on request from the corresponding author.
